# Author Correction: Sexual morph specialisation in a trioecious nematode balances opposing selective forces

**DOI:** 10.1038/s41598-022-14564-5

**Published:** 2022-06-14

**Authors:** Sally Adams, Prachi Pathak, Maike Kittelmann, Alun R. C. Jones, Eamonn B. Mallon, Andre Pires‑daSilva

**Affiliations:** 1 School of Life Sciences, University of Warwick, Coventry, CV4 7AL UK; 2 Department of Biological and Medical Sciences, Oxford Brookes University, Headington Campus, Oxford, OX3 0BP UK; 3 Department of Genetics and Genome Biology, University of Leicester, University Road, Leicester, LE1 7RH UK

Correction to: *Scientific Reports* 10.1038/s41598-022-09900-8, published on 17 April 2022

The original version of this Article contained an error in the spelling of the author Maike Kittelmann which was incorrectly given as Maike Kittelman.

The original Article and accompanying Supplementary Information file have been corrected.

